# Crystal structure determination and analyses of Hirshfeld surface, crystal voids, inter­molecular inter­action energies and energy frameworks of 1-benzyl-4-(methyl­sulfan­yl)-3a,7a-di­hydro-1*H*-pyrazolo­[3,4-*d*]pyrimidine

**DOI:** 10.1107/S2056989024005954

**Published:** 2024-06-25

**Authors:** Nour El Hoda Mustaphi, Amina Chlouchi, Mohamed El Hafi, Joel T. Mague, Tuncer Hökelek, Hanae El Monfalouti, Amal Haoudi, Ahmed Mazzah

**Affiliations:** a Organic Chemistry Catalysis and Environmental Laboratory Higher National School of Chemistry Ibn Tofail University Kenitra Morocco; b Faculty of Medicine and Pharmacy, Mohammed First University, Oujda, Morocco; c Laboratory of Heterocyclic Organic Chemistry URAC 21 Pharmacochemistry Competence Center Av Ibn Battouta BP 1014 Faculty of Sciences Mohammed V University in Rabat Morocco; d Department of Chemistry, Tulane University, New Orleans, LA 70118, USA; e Department of Physics, Hacettepe University, 06800 Beytepe, Ankara, Türkiye; f Laboratory of Plant Chemistry Organic and Bioorganic Synthesis Faculty of Sciences Mohammed V University in Rabat 4 Avenue Ibn Battouta BP 1014 RP Morocco; g Laboratory of Applied Organic Chemistry, Sidi Mohamed Ben Abdellah University, Faculty Of Science And Technology, Road Immouzer, BP 2202 Fez, Morocco; h Science and Technology of Lille USR 3290, Villeneuve d’Ascq cedex, France; Vienna University of Technology, Austria

**Keywords:** crystal structure, pyrazolo­pyrimidine, sulfide, hydrogen bond, C—H⋯π(ring) inter­action

## Abstract

The pyrazolo­pyrimidine moiety in the title mol­ecule, C_13_H_12_N_4_S, is planar with the methyl­sulfanyl substituent lying essentially in the same plane. The benzyl group is rotated well out of this plane by 73.64 (6)°, giving the mol­ecule an approximate *L* shape. In the crystal, C—H⋯π(ring) inter­actions and C—H⋯S hydrogen bonds form tubes extending along the *a* axis. Furthermore, there are π–π inter­actions between parallel phenyl rings with centroid-to-centroid distances of 3.8418 (12) Å. A Hirshfeld surface analysis of the crystal structure indicates that the most important contributions to the crystal packing are from H⋯H (47.0%), H⋯N/N⋯H (17.6%) and H⋯C/C⋯H (17.0%) inter­actions. The volume of the crystal voids and the percentage of free space were calculated to be 76.45 Å^3^ and 6.39%, showing that there is no large cavity in the crystal packing. Evaluation of the electrostatic, dispersion and total energy frameworks indicate that the cohesion of the crystal structure is dominated by the dispersion energy contributions.

## Abstract 2

In the title mol­ecule, the pyrazolo­pyrimidine moiety is planar with the methyl­sulfanyl substituent lying essentially in the same plane, whereas the benzyl group is rotated well out of this plane giving the mol­ecule an approximate *L* shape.

## 1. Chemical context

The chemistry of heterocyclic compounds has attracted increasing inter­est in recent decades, driven by the therapeutic potential of many of these compounds, particularly those containing nitro­gen. Notably, nitro­gen heterocycles have emerged as promising candidates for bioactive mol­ecules (Irrou *et al.*, 2022 ▸; Sebbar *et al.*, 2016 ▸). Among these, pyrazolo­[3,4-*d*]pyrimidine stands out as an important compound, with its derivatives exhibiting various pharmacological properties (Severina *et al.*, 2016 ▸). They are widely used in pharmaceutical research for their anti-tumour (Kandeel *et al.*, 2012 ▸), anti-inflammatory (El-Tombary, 2013 ▸), anti­microbial (Bakavoli *et al.*, 2010 ▸), anti­oxidant (El-Mekabaty, 2015 ▸), anti­convulsant (Severina *et al.*, 2016 ▸) and anti­cancer (Maher *et al.*, 2019 ▸) properties. Additionally, pyrazolo­pyrimidines have been shown to treat Alzheimer’s disease (Zhang *et al.*, 2018 ▸), human leukaemia (HL-60) (Song *et al.*, 2011 ▸) and exert potent activity against viruses of herpes (Gudmundsson *et al.*, 2009 ▸).
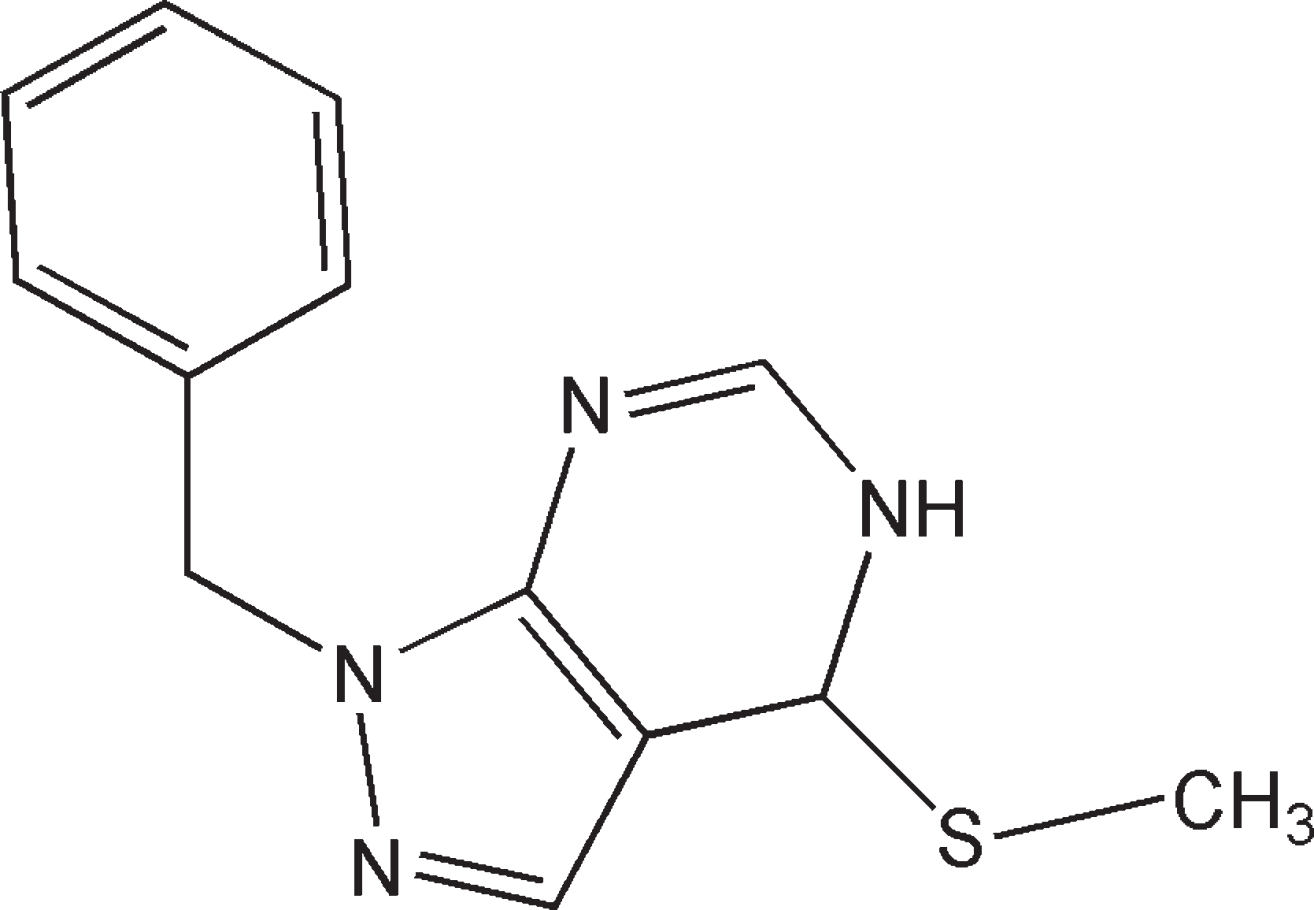


Continuing our research in this area, we synthesized the title compound, 1-benzyl-4-(methyl­sulfan­yl)-3a,7a-di­hydro-1*H*-pyrazolo­[3,4-*d*]pyrimidine, (I), and carried out its crystal-structure determination, as well as analyses of the Hirshfeld surface, crystal voids, inter­molecular inter­action energies and energy frameworks.

## 2. Structural commentary

**Figure 1:**
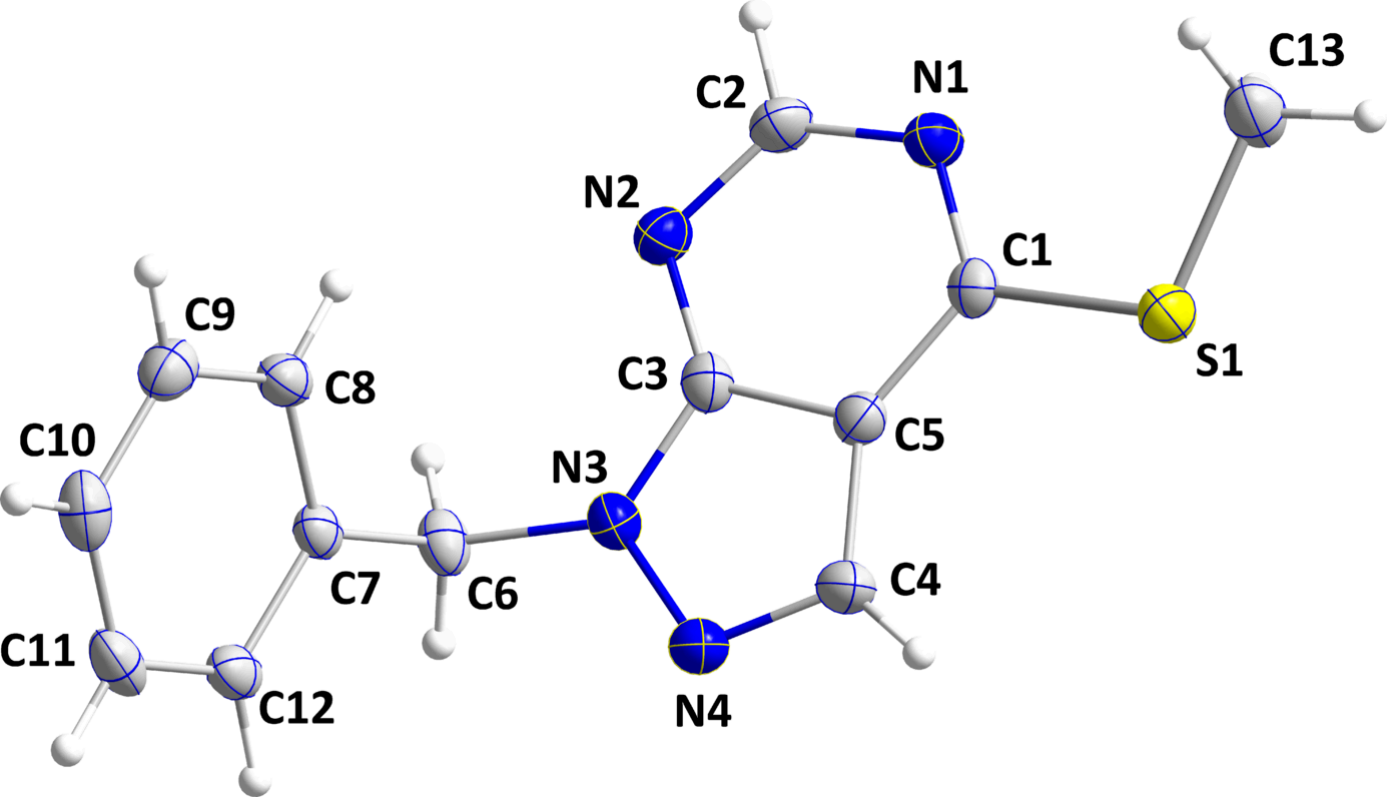
The mol­ecular structure of (I) with the labelling scheme and displacement ellipsoids drawn at the 50% probability level.

The pyrazolo­pyrimidine moiety of (I) is essentially planar (root-mean-square deviation = 0.0046 Å), and the C7–C12 phenyl ring is inclined to this plane by 73.64 (6)°, giving the mol­ecule an approximate *L* shape (Fig. 1 ▸). The methyl­sulfanyl substituent lies in the mean plane of the pyrazolo­pyrimidine moiety, as indicated by the N1—C1—S1—C13 torsion angle of −0.32 (18)°. All bond lengths and angles in this mol­ecule appear to be characteristic.

## 3. Supra­molecular features

**Figure 2:**
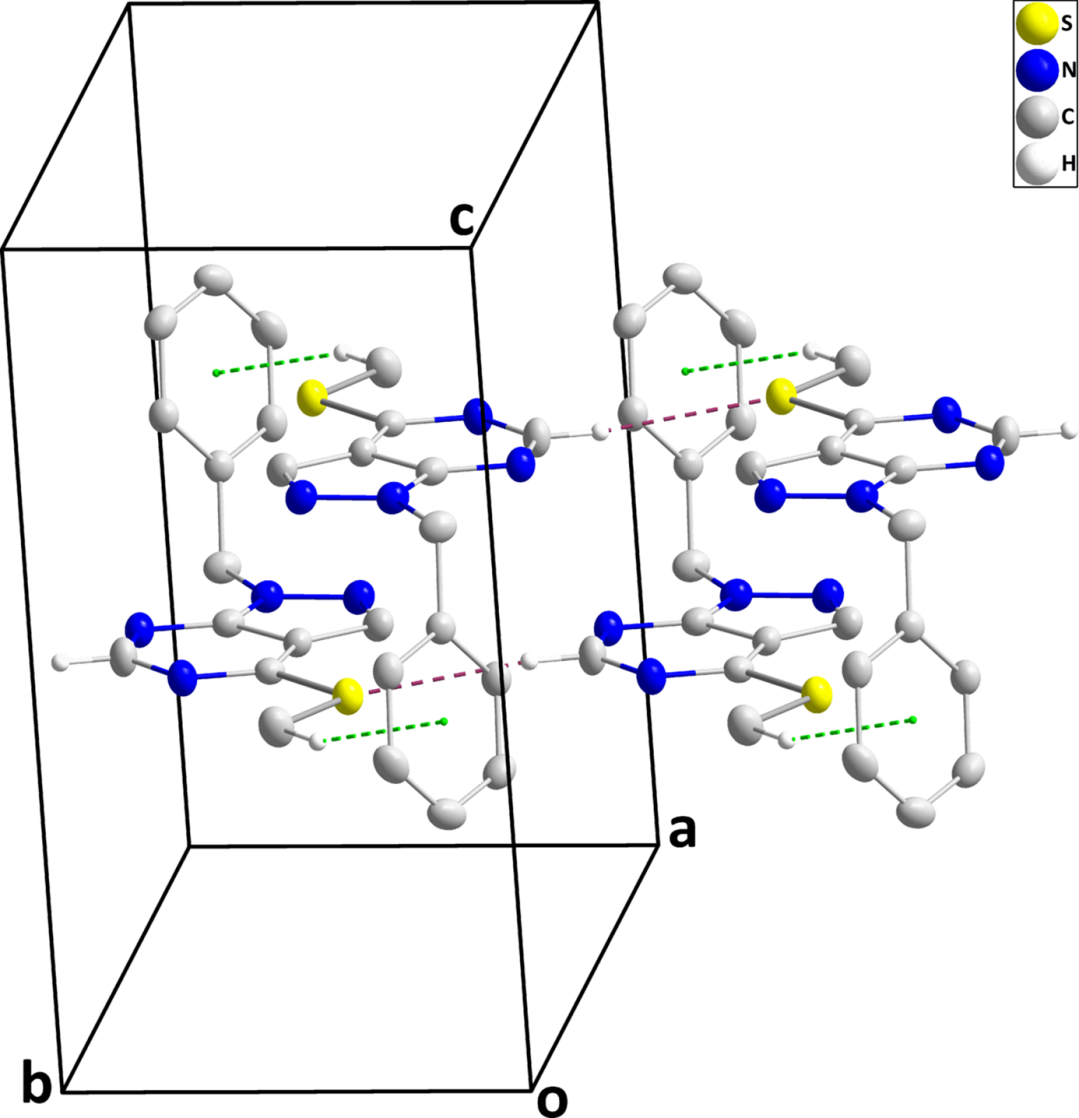
Detail of a portion of one tube with C—H⋯S hydrogen-bonding inter­actions and C—H⋯π(ring) inter­actions shown, respectively, by purple and green dashed lines.

**Figure 3:**
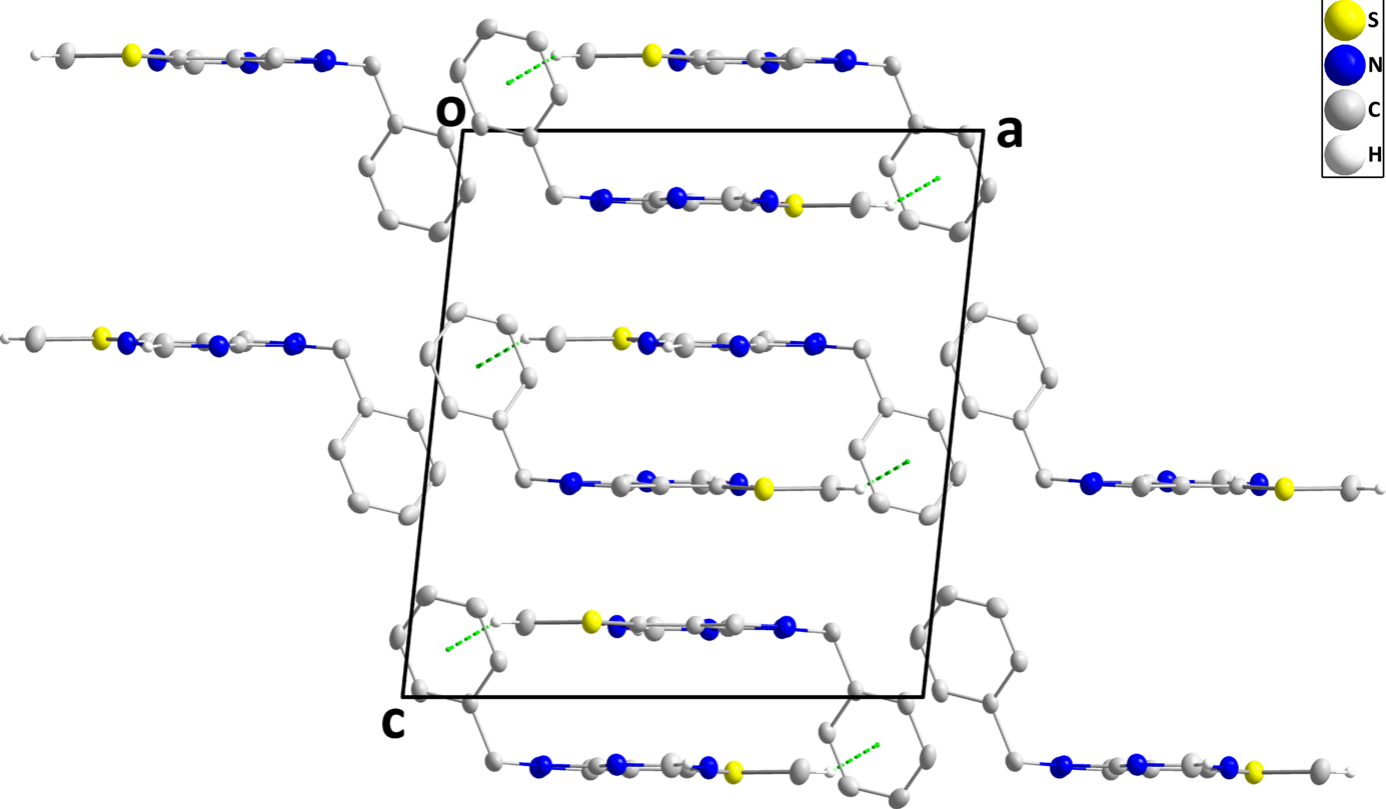
Packing giving an end view of three tubes seen along the *b* axis with C—H⋯π(ring) inter­actions shown as dashed lines.

In the crystal of (I), inversion dimers are formed by C13—H13*C*⋯*Cg*3^ii^ inter­actions (*Cg*3 is the centroid of the C7–C12 phenyl ring). Through additional C—H⋯S hydrogen bonds, the dimers are connected into rectangular tubes extending parallel to the *a* axis (Table 1 ▸, Fig. 2 ▸). The tubes are stacked along the *c* axis by van der Waals contacts between them (Fig. 3 ▸). Furthermore, there are *Cg*3–*Cg*3^i^ inter­actions between parallel phenyl rings with a centroid-to-centroid distances of 3.8418 (12) Å [α = 0.03 (10)°; symmetry code: (i) −*x*, −*y*, 1 − *z*].

## 4. Hirshfeld surface analysis

**Figure 4:**
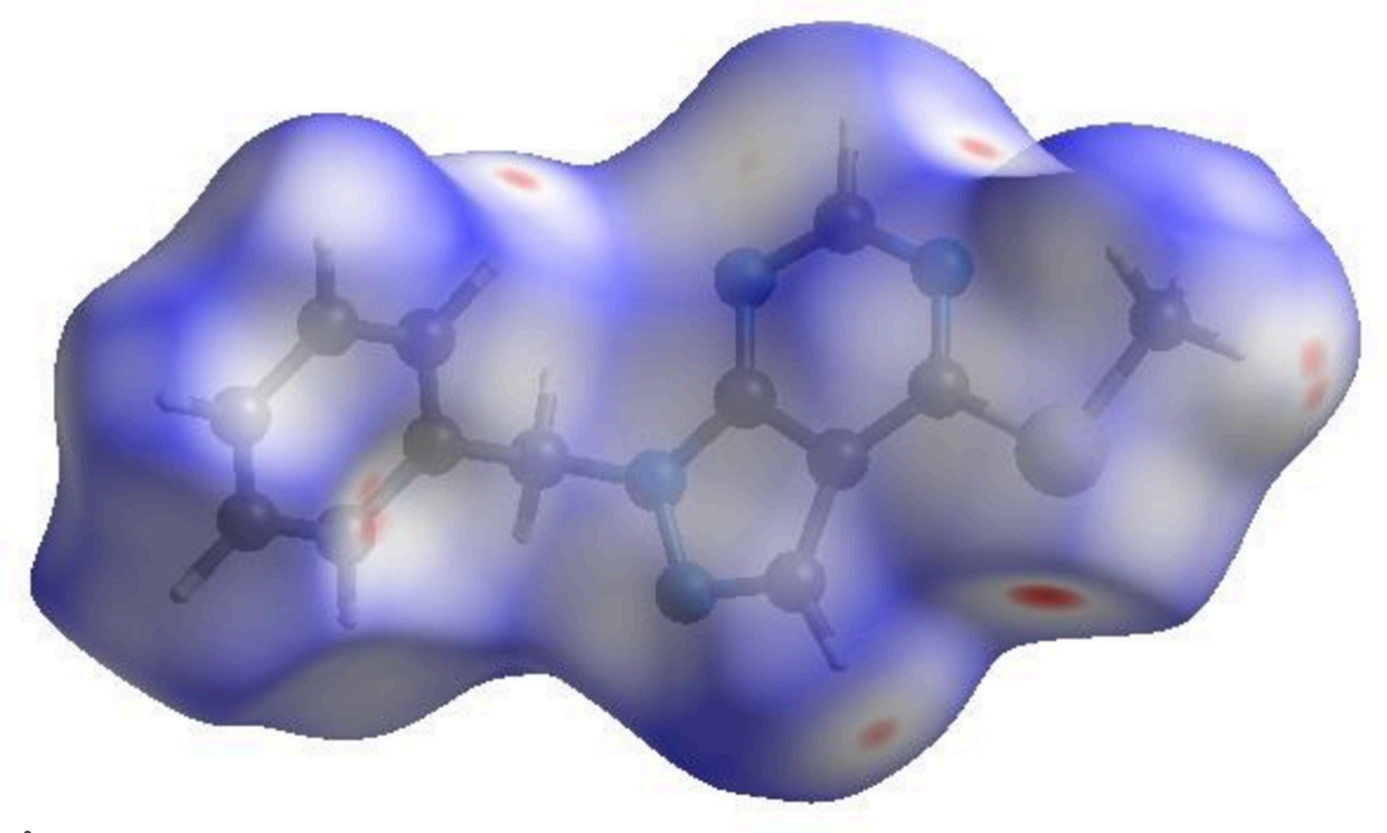
View of the three-dimensional Hirshfeld surface of the title compound plotted over *d*_norm_.

**Figure 5:**
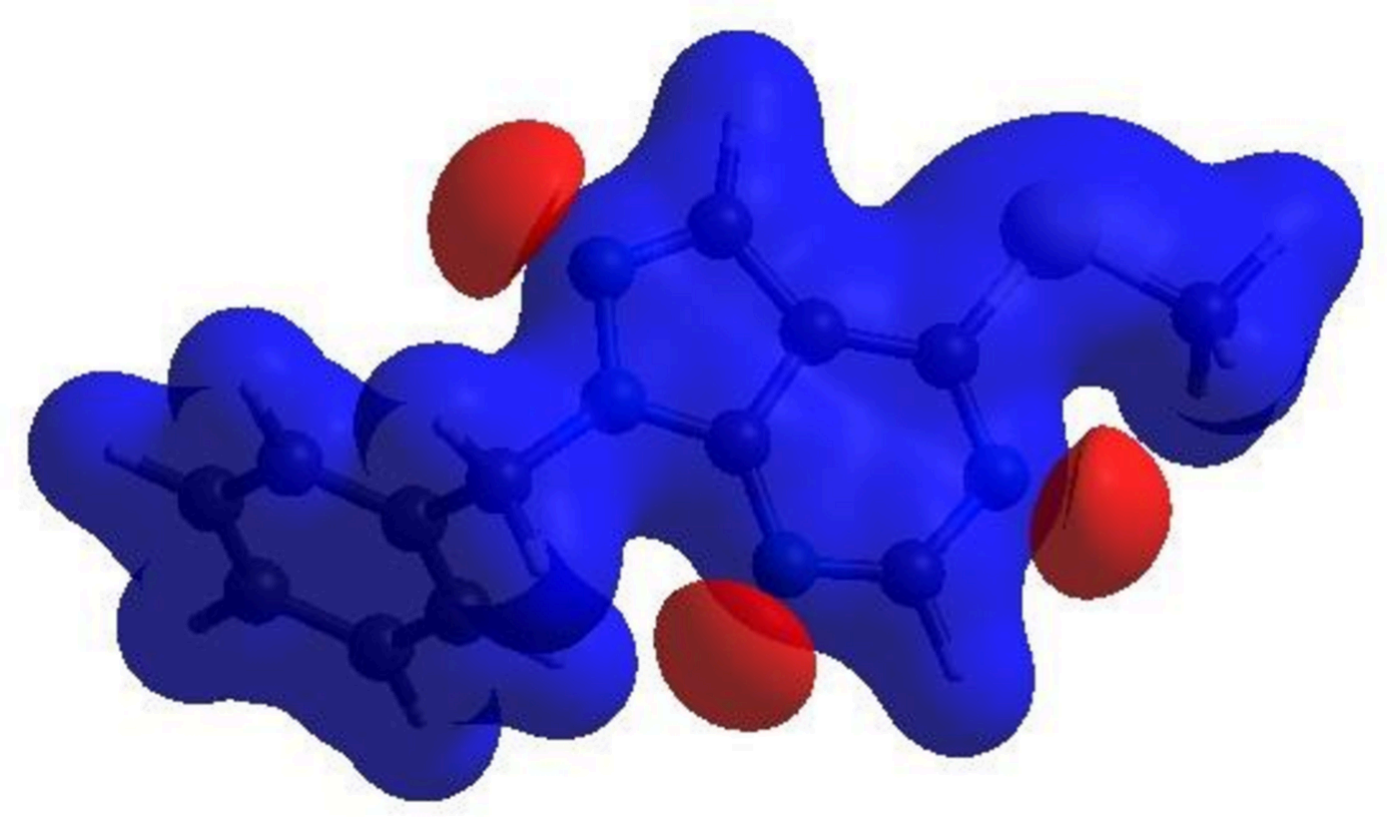
View of the three-dimensional Hirshfeld surface of the title compound plotted over electrostatic potential energy using the STO-3 G basis set at the Hartree–Fock level of theory. Hydrogen-bond donors and acceptors are shown as blue and red regions around the atoms corresponding to positive and negative potentials, respectively.

**Figure 6:**
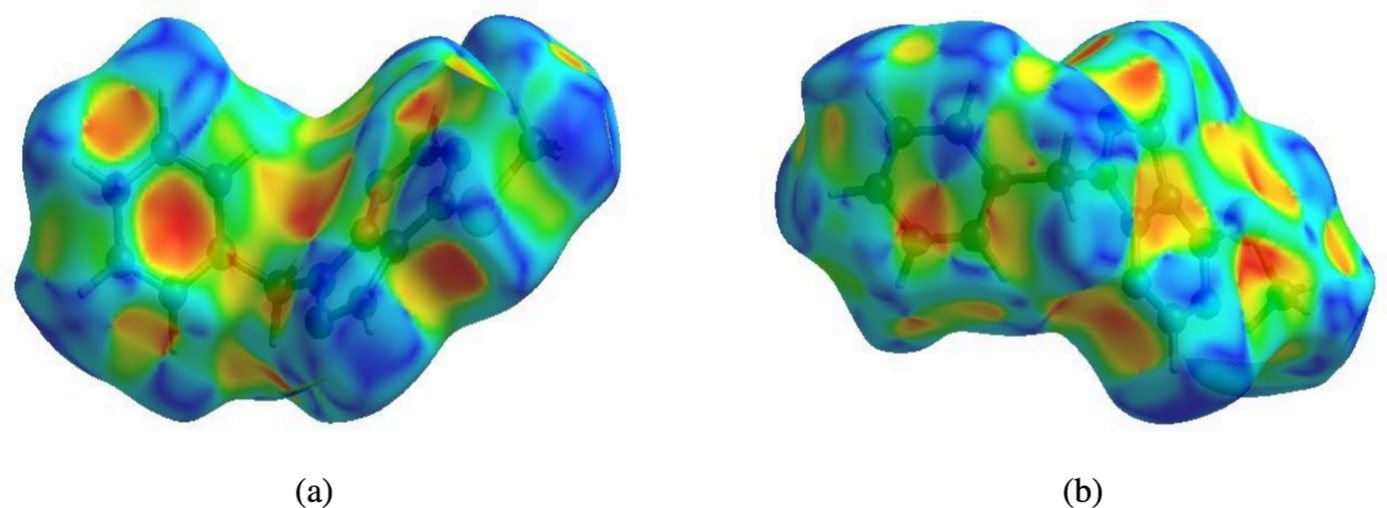
Hirshfeld surface of the title compound plotted over shape-index for two orientations.

In order to visualize the inter­molecular inter­actions in the crystal of (I), a Hirshfeld surface (HS) analysis (Hirshfeld, 1977 ▸; Spackman & Jayatilaka, 2009 ▸) was carried out by using *CrystalExplorer* (Spackman *et al.*, 2021 ▸). In the HS plotted over *d*_norm_ (Fig. 4 ▸), the white surface indicates contacts with distances equal to the sum of van der Waals radii, and the red and blue areas indicate distances shorter (in close contact) or longer (distant contact) than the van der Waals radii, respectively (Venkatesan *et al.*, 2016 ▸). The bright-red spots indicate their roles as the respective donors and/or acceptors; they also appear as blue and red regions corresponding to positive and negative potentials on the HS mapped over electrostatic potential (Spackman *et al.*, 2008 ▸; Jayatilaka *et al.*, 2005 ▸), as shown in Fig. 5 ▸. The blue regions indicate positive electrostatic potential (hydrogen-bond donors), while the red regions indicate negative electrostatic potential (hydrogen-bond acceptors). The π–π stacking and C—H⋯π inter­actions were further visualized by the shape-index surface. This surface can be used to identify characteristic packing modes, in particular, planar stacking arrangements and the presence of aromatic stacking inter­actions. In this regard, the shape-index represents the C—H⋯π inter­actions as ‘red *p*-holes’, which are related to the electron ring inter­actions between the CH groups with the centroid of the aromatic rings of neighbouring mol­ecules. Fig. 6 ▸*a* clearly suggests that there are C—H⋯π inter­actions in (I), and π–π stacking is indicated by the presence of adjacent red and blue triangles (Fig. 6 ▸*b*).

**Figure 7:**
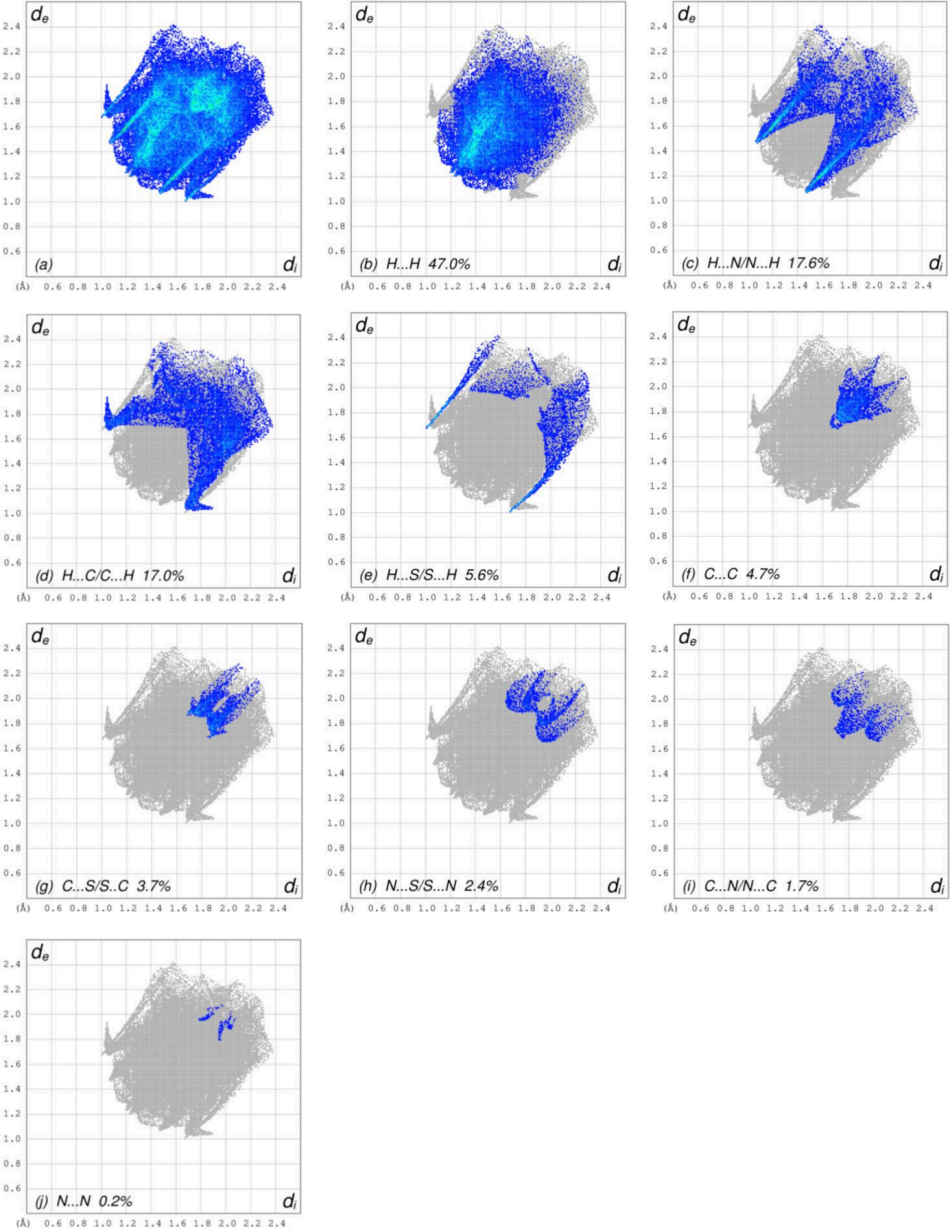
The full two-dimensional fingerprint plots for the title compound, showing (*a*) all inter­actions, and delineated into (*b*) H⋯H, (*c*) H⋯N/N⋯H, (*d*) H⋯C/C⋯H (*e*) H⋯S/S⋯H, (*f*) C⋯C, (*g*) C⋯S/S⋯C, (*h*) N⋯S/S⋯N, (i) C⋯N/N⋯C and (*j*) N⋯N inter­actions. The *d*_i_ and *d*_e_ values are the closest inter­nal and external distances (in Å) from given points on the Hirshfeld surface.

The overall two-dimensional fingerprint plot, Fig. 7 ▸*a*, and those delineated into H⋯H, H⋯N/N⋯H, H⋯C/C⋯H, H⋯S/S⋯H, C⋯C, C⋯S/S⋯C, N⋯S/S⋯N, C⋯N/N⋯C and N⋯N contacts (McKinnon *et al.*, 2007 ▸) are illustrated in Fig. 7 ▸*b*–*j*, respectively, together with their relative contributions to the Hirshfeld surface. The most important inter­action is H⋯H, contributing 47.0% to the overall crystal packing, which is reflected in Fig. 7 ▸*b* as widely scattered points of high density due to the large hydrogen content of the mol­ecule with the tip at *d*_e_ = *d*_i_ = 1.20 Å. The symmetrical pair of spikes resulting in the fingerprint plot delineated into H⋯N/N⋯H contacts (Fig. 7 ▸*c*) with a 17.6% contribution to the HS has the tips at *d*_e_ + *d*_i_ = 2.52 Å. In the presence of C—H⋯π inter­actions (Table 1 ▸, Fig. 6 ▸), the H⋯C/C⋯H contacts, contributing 17.0% to the overall crystal packing, are reflected in Fig. 7 ▸*d* with the tips at *d*_e_ + *d*_i_ = 2.73 Å. The H⋯S/S⋯H contacts (Fig. 7 ▸*e*) contribute 5.6% to the HS, and their symmetrical pair of spikes has the tips at *d*_e_ + *d*_i_ = 2.68 Å. The C⋯C contacts (Fig. 7 ▸*f*) have an arrow-shaped distribution of points, contributing 4.7% to the HS, with the tip at *d*_e_ = *d*_i_ = 1.68 Å. The symmetrical pairs of C⋯S/S⋯C (Fig. 7 ▸*g*) and N⋯S/S⋯N (Fig. 7 ▸*h*) contacts contribute 3.7% and 2.4% to the HS, and they are observed with the tips at *d*_e_ + *d*_i_ = 3.58 Å and *d*_e_ + *d*_i_ = 3.61 Å, respectively. Finally, the C⋯N/N⋯C (Fig. 7 ▸*i*) and N⋯N (Fig. 7 ▸*j*) contacts, with 1.7% and 0.2% contributions to the HS, have very low abundance.

**Figure 8:**
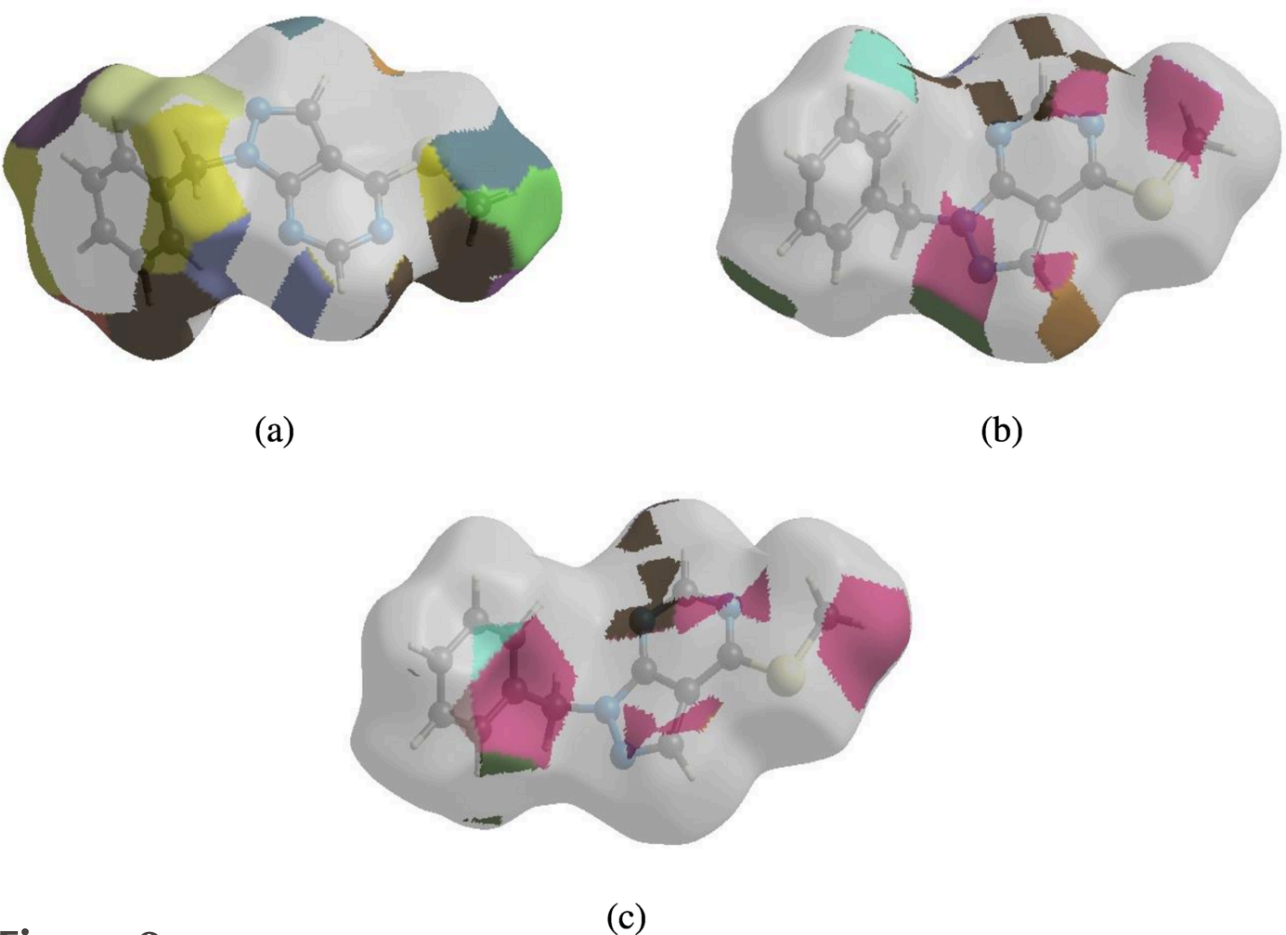
The Hirshfeld surface representations with the fragment patch plotted onto the surface for (*a*) H⋯H, (*b*) H⋯N/N⋯H and (*c*) H⋯C/C⋯H inter­actions.

The nearest neighbour environment of a mol­ecule can be determined from the colour patches on the HS based on how close to other mol­ecules they are. The Hirshfeld surface representations with the function *d*_norm_ plotted onto the surface are shown for the H⋯H, H⋯N/N⋯H and H⋯C/C⋯H inter­actions in Fig. 8 ▸*a*–*c*, respectively. The Hirshfeld surface analysis confirms the importance of H-atom contacts in establishing the packing. The large number of H⋯H, H⋯N/N⋯H and H⋯C/C⋯H inter­actions suggest that van der Waals inter­actions and hydrogen bonding play the major roles in the crystal packing (Hathwar *et al.*, 2015 ▸).

## 5. Crystal voids

**Figure 9:**
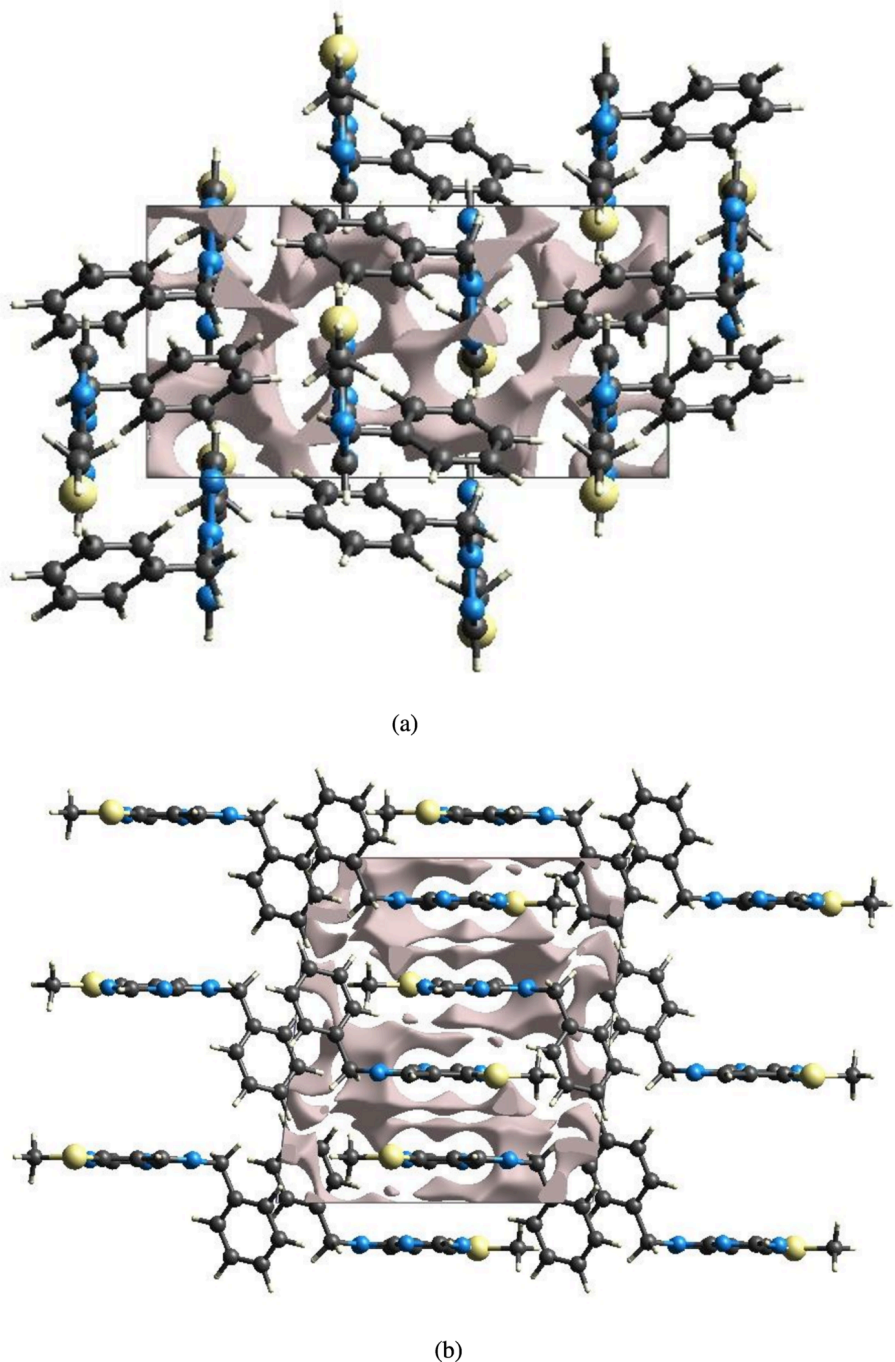
Graphical views of voids in the crystal packing of (I) (*a*) along the *a* axis and (*b*) along the *b* axis. The grey shaded areas represent the filled regions (electron densities), while the colourless regions represent the crystal voids (free spaces).

The strength of the crystal packing is important for determining the response to an applied mechanical force. For checking the mechanical stability of the crystal, a void analysis was performed by adding up the electron densities of the spherically symmetric atoms comprised in the asymmetric unit (Turner *et al.*, 2011 ▸). The void surface is defined as an isosurface of the procrystal electron density and is calculated for the whole unit cell where the void surface meets the boundary of the unit cell and capping faces are generated to create an enclosed volume. The volume of the crystal voids (Fig. 9 ▸*a*,*b*) and the percentage of free space in the unit cell are calculated as 76.45 Å^3^ and 6.39%, respectively. Thus, the crystal packing appears compact and the mechanical stability should be substantial.

## 6. Inter­action energy calculations and energy frameworks

**Figure 10:**
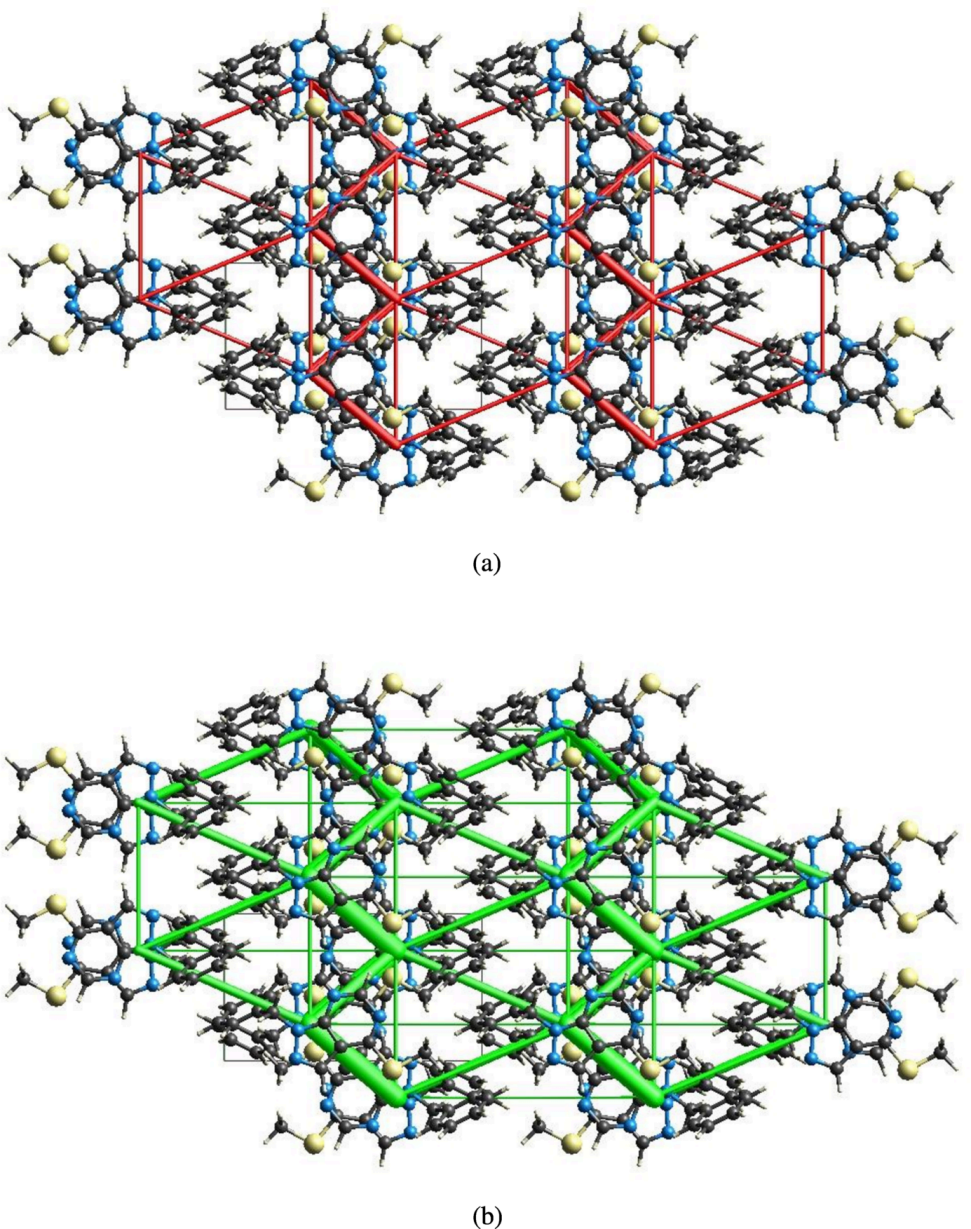
The energy frameworks for a cluster of mol­ecules of (I) viewed down the *c* axis showing (*a*) electrostatic energy and (*b*) dispersion energy diagrams. The cylindrical radius is proportional to the relative strength of the corresponding energies and they were adjusted to the same scale factor of 80 with cut-off value of 5 kJ mol^−1^ within 2×2×2 unit cells.

The inter­molecular inter­action energies were calculated using the CE–B3LYP/6–31G(d,p) energy model available in *CrystalExplorer* (Spackman *et al.*, 2021 ▸), where a cluster of mol­ecules is generated by applying crystallographic symmetry operations with respect to a selected central mol­ecule within the radius of 3.8 Å by default (Turner *et al.*, 2014 ▸). The total inter­molecular energy (*E*_tot_) is the sum of electrostatic (*E*_ele_), polarization (*E*_pol_), dispersion (*E*_dis_) and exchange-repulsion (*E*_rep_) energies (Turner *et al.*, 2015 ▸) with scale factors of 1.057, 0.740, 0.871 and 0.618, respectively (Mackenzie *et al.*, 2017 ▸). Hydrogen-bonding inter­action energies (in kJ mol^−1^) were calculated to be −30.3(*E*_ele_), −3.6 (*E*_pol_), −74.7 (*E*_dis_), 70.9 (*E*_rep_) and −55.9 (*E*_tot_) for the C2—H2⋯S1 hydrogen-bonding inter­action. Energy frameworks combine the calculation of inter­molecular inter­action energies with a graphical representation of their magnitude (Turner *et al.*, 2015 ▸). Energies between mol­ecular pairs are represented as cylinders joining the centroids of pairs of mol­ecules with the cylinder radius proportional to the relative strength of the corresponding inter­action energy. Energy frameworks were constructed for *E*_ele_ (red cylinders) and *E*_dis_ (green cylinders) (Fig. 10 ▸*a*,*b*). The evaluation of the electrostatic, dispersion and total energy frameworks indicate that the stabilization is dominated *via* the dispersion energy contributions in the crystal structure of (I).

## 7. Database survey

**Figure 11:**
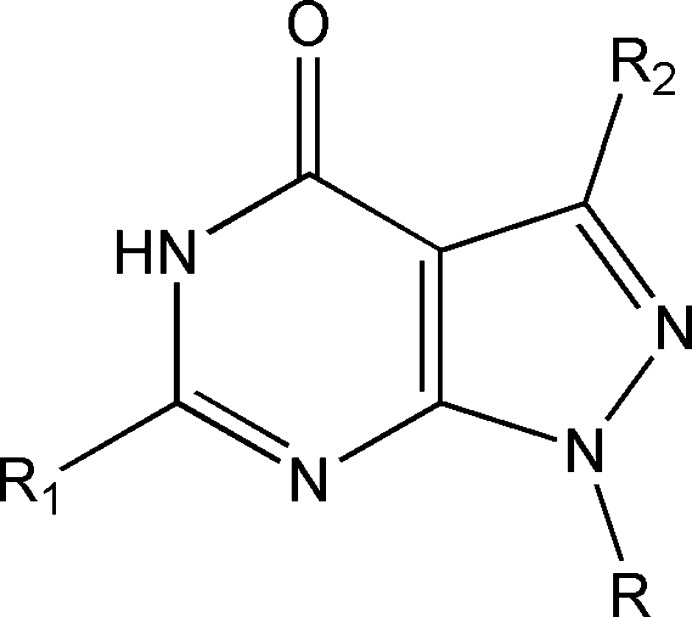
Scheme used for the database search.

A search of the Cambridge Structural Database (CSD, updated to March 2024; Groom *et al.*, 2016 ▸) using the search fragment detailed in Fig. 11 ▸ (*R* = C—CH, C—C—OH; *R*_1_ = *R*_2_ = nothing) identified eleven relevant hits. These structures include *R* = *t*-Bu, *R*_2_ = H, *R*_1_ = Ph (RULHEN; Liu *et al.*, 2015 ▸), *p*-anis (QIBVIH; Tan *et al.*, 2007 ▸); *R*_2_ = H, *R* = *i*-Pr, *R*_1_ = cyclo­butane­carboxamido (QIBVON; Tan *et al.*, 2007 ▸), *R* = *n*-Bu, *R*_1_ = benzamido (QIBWAA; Tan *et al.*, 2007 ▸), *R* = 3-phenyl­propyl, *R*_1_= CH_3_S (IFICUV; Avasthi *et al.*, 2002 ▸), *R* = 2-chloro­ethyl, *R*_1_= H (XAZRAT; Khazi *et al.*, 2012 ▸); *R* = 1-β-d-ribo­furanosyl, *R*_1_ = H, *R*_2_ = OMe (FOVHIH; Anderson *et al.*, 1986 ▸), *R*_1_= NH_2_, *R*" = H (YOMJIW; Ren *et al.*, 2019 ▸); *R* = 2-de­oxy-β-d-*erythro*-pento­furanosyl, *R*_1_ = NH_2_, *R*_2_ = Br (HIPPAX; Seela *et al.*, 1999 ▸), *R*_2_ = I (HIPPEB; Seela *et al.*, 1999 ▸); *R* = 2-de­oxy-2-fluoro-β-d-arabino­furanosyl, *R*_1_= NH_2_, *R*_2_= Br (EJEJUY; He *et al.*, 2003 ▸). Analysis of the mol­ecular geometries revealed that while the pyrazolo­pyrimidine unit remained essentially planar as in the mol­ecule of (I), the diversity of substituents in these related structures and the presence of additional hydrogen bonding results in distinctly different crystal packings.

## 8. Synthesis and crystallization

A catalytic amount of tetra-*n*-butyl­ammonium bromide (0.33 mmol) was added to a solution of 1-benzyl-1*H*-pyrazolo­[3,4-d]pyrimidine-4(5*H*)-thione (10 mmol), iodo­methane (10 mmol) and potassium carbonate (6.51 mmol) in di­methyl­formamide (DMF, 40 ml). The mixture was stirred for 24 h. The solid material was removed by filtration and the solvent evaporated *in vacuo*. The resulting colourless solid product was purified by recrystallization from ethanol. Yield: 82%.

## 9. Refinement

Crystal data, data collection and structure refinement details are summarized in Table 2 ▸. Hydrogen atoms were located in difference-Fourier maps and were refined freely.

## Supplementary Material

Crystal structure: contains datablock(s) global, I. DOI: 10.1107/S2056989024005954/wm5721sup1.cif

Structure factors: contains datablock(s) I. DOI: 10.1107/S2056989024005954/wm5721Isup2.hkl

Supporting information file. DOI: 10.1107/S2056989024005954/wm5721Isup3.cdx

Supporting information file. DOI: 10.1107/S2056989024005954/wm5721Isup4.cml

CCDC reference: 2363971

Additional supporting information:  crystallographic information; 3D view; checkCIF report

## Figures and Tables

**Table 1:** Hydrogen-bond geometry (Å, °) *Cg*3 is the centroid of the C7–C12 phenyl ring.

*D*—H⋯*A*	*D*—H	H⋯*A*	*D*⋯*A*	*D*—H⋯*A*
C2—H2⋯S1^i^	0.97 (3)	2.81 (3)	3.781 (2)	174 (2)
C13—H13*C*⋯*Cg*3^ii^	1.02 (4)	2.49 (4)	3.455 (2)	157 (3)

Symmetry codes: (i) 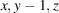; (ii) 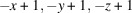.

**Table 2:** Experimental details

Crystal data	
Chemical formula	C_13_H_12_N_4_S
*M*_r_	256.33
Crystal system, space group	Monoclinic, *P*2_1_/*c*
Temperature (K)	150
*a*, *b*, *c* (Å)	12.4617 (5), 7.0766 (3), 13.6500 (5)
β (°)	96.085 (1)
*V* (Å^3^)	1196.96 (8)
*Z*	4
Radiation type	Cu *K*α
μ (mm^−1^)	2.29
Crystal size (mm)	0.26 × 0.22 × 0.16

Data collection	
Diffractometer	Bruker D8 VENTURE PHOTON 100 CMOS
Absorption correction	Multi-scan (*SADABS*; Krause *et al.*, 2015 ▸)
*T*_min_, *T*_max_	0.59, 0.72
No. of measured, independent and observed [*I* > 2σ(*I*)] reflections	8877, 2388, 2292
*R*_int_	0.026
(sin θ/λ)_max_ (Å^−1^)	0.626

Refinement	
*R*[*F*^2^ > 2σ(*F*^2^)], *wR*(*F*^2^), *S*	0.049, 0.135, 1.10
No. of reflections	2388
No. of parameters	212
H-atom treatment	All H-atom parameters refined
Δρ_max_, Δρ_min_ (e Å^−3^)	0.68, −0.42

Computer programs: *APEX3* and *SAINT* (Bruker, 2016 ▸), *SAINT* (Bruker, 2016 ▸), *SHELXT/5* (Sheldrick, 2015*a* ▸), *SHELXL2018/3* (Sheldrick, 2015*b* ▸), *DIAMOND* (Brandenburg & Putz, 2012 ▸) and *SHELXTL* (Sheldrick, 2008 ▸).

## supplementary crystallographic information

### 1-Benzyl-4-(methylsulfanyl)-3a,7a-dihydro-1H-pyrazolo[3,4-d]pyrimidine Crystal data

**Table:**

C_13_H_12_N_4_S	*F*(000) = 536
*M**_r_* = 256.33	*D*_x_ = 1.422 Mg m^−^^3^
Monoclinic, *P*2_1_/*c*	Cu *K*α radiation, λ = 1.54178 Å
*a* = 12.4617 (5) Å	Cell parameters from 7875 reflections
*b* = 7.0766 (3) Å	θ = 6.3–74.7°
*c* = 13.6500 (5) Å	µ = 2.29 mm^−^^1^
β = 96.085 (1)°	*T* = 150 K
*V* = 1196.96 (8) Å^3^	Block, colourless
*Z* = 4	0.26 × 0.22 × 0.16 mm

### 1-Benzyl-4-(methylsulfanyl)-3a,7a-dihydro-1H-pyrazolo[3,4-d]pyrimidine Data collection

**Table:**

Bruker D8 VENTURE PHOTON 100 CMOS diffractometer	2388 independent reflections
Radiation source: INCOATEC IµS micro–focus source	2292 reflections with *I* > 2σ(*I*)
Mirror monochromator	*R*_int_ = 0.026
Detector resolution: 10.4167 pixels mm^-1^	θ_max_ = 74.7°, θ_min_ = 6.5°
ω scans	*h* = −15→14
Absorption correction: multi-scan (*SADABS*; Krause *et al.*, 2015)	*k* = −8→8
*T*_min_ = 0.59, *T*_max_ = 0.72	*l* = −15→17
8877 measured reflections	

### 1-Benzyl-4-(methylsulfanyl)-3a,7a-dihydro-1H-pyrazolo[3,4-d]pyrimidine Refinement

**Table:**

Refinement on *F*^2^	Secondary atom site location: difference Fourier map
Least-squares matrix: full	Hydrogen site location: difference Fourier map
*R*[*F*^2^ > 2σ(*F*^2^)] = 0.049	All H-atom parameters refined
*wR*(*F*^2^) = 0.135	*w* = 1/[σ^2^(*F*_o_^2^) + (0.0792*P*)^2^ + 0.9126*P*] where *P* = (*F*_o_^2^ + 2*F*_c_^2^)/3
*S* = 1.10	(Δ/σ)_max_ = 0.001
2388 reflections	Δρ_max_ = 0.68 e Å^−^^3^
212 parameters	Δρ_min_ = −0.42 e Å^−^^3^
0 restraints	Extinction correction: *SHELXL2018/3* (Sheldrick, 2015*b*), Fc^*^=kFc[1+0.001xFc^2^λ^3^/sin(2θ)]^-1/4^
Primary atom site location: dual	Extinction coefficient: 0.0175 (15)

### 1-Benzyl-4-(methylsulfanyl)-3a,7a-dihydro-1H-pyrazolo[3,4-d]pyrimidine Special details

**Table:**

Geometry. All esds (except the esd in the dihedral angle between two l.s. planes) are estimated using the full covariance matrix. The cell esds are taken into account individually in the estimation of esds in distances, angles and torsion angles; correlations between esds in cell parameters are only used when they are defined by crystal symmetry. An approximate (isotropic) treatment of cell esds is used for estimating esds involving l.s. planes.
Refinement. Refinement of F^2^ against ALL reflections. The weighted R-factor wR and goodness of fit S are based on F^2^, conventional R-factors R are based on F, with F set to zero for negative F^2^. The threshold expression of F^2^ > 2sigma(F^2^) is used only for calculating R-factors(gt) etc. and is not relevant to the choice of reflections for refinement. R-factors based on F^2^ are statistically about twice as large as those based on F, and R- factors based on ALL data will be even larger.

### 1-Benzyl-4-(methylsulfanyl)-3a,7a-dihydro-1H-pyrazolo[3,4-d]pyrimidine Fractional atomic coordinates and isotropic or equivalent isotropic displacement parameters (Å^2^)

**Table:**

	*x*	*y*	*z*	*U*_iso_*/*U*_eq_	
S1	0.65190 (4)	0.56176 (7)	0.63280 (4)	0.0270 (2)	
N1	0.60237 (13)	0.1966 (2)	0.62462 (13)	0.0254 (4)	
N2	0.42503 (15)	0.0580 (2)	0.61920 (13)	0.0250 (4)	
N3	0.28464 (13)	0.2921 (2)	0.62089 (12)	0.0236 (4)	
N4	0.27715 (14)	0.4851 (3)	0.62427 (13)	0.0262 (4)	
C1	0.56283 (15)	0.3718 (3)	0.62822 (13)	0.0202 (4)	
C2	0.53049 (18)	0.0519 (3)	0.62047 (17)	0.0286 (5)	
H2	0.561 (2)	−0.074 (4)	0.618 (2)	0.040 (8)*	
C3	0.38820 (15)	0.2371 (3)	0.62210 (13)	0.0217 (4)	
C4	0.37631 (17)	0.5524 (3)	0.62805 (15)	0.0250 (4)	
H4	0.3872 (18)	0.676 (4)	0.6326 (16)	0.016 (5)*	
C5	0.45191 (16)	0.4014 (3)	0.62684 (14)	0.0213 (4)	
C6	0.18860 (16)	0.1711 (3)	0.61404 (14)	0.0254 (4)	
H6A	0.213 (2)	0.050 (4)	0.6369 (18)	0.024 (6)*	
H6B	0.144 (2)	0.215 (4)	0.6594 (18)	0.024 (6)*	
C7	0.13101 (14)	0.1627 (3)	0.51110 (13)	0.0204 (4)	
C8	0.17719 (16)	0.0661 (3)	0.43709 (15)	0.0232 (4)	
H8	0.242 (2)	0.004 (4)	0.4507 (16)	0.021 (5)*	
C9	0.12422 (18)	0.0575 (3)	0.34245 (16)	0.0271 (5)	
H9	0.156 (2)	−0.003 (4)	0.295 (2)	0.038 (7)*	
C10	0.02500 (17)	0.1456 (3)	0.32115 (16)	0.0293 (5)	
H10	−0.004 (2)	0.137 (4)	0.264 (2)	0.041 (8)*	
C11	−0.02157 (16)	0.2423 (3)	0.39411 (16)	0.0290 (5)	
H11	−0.082 (2)	0.299 (4)	0.3801 (19)	0.033 (7)*	
C12	0.03167 (15)	0.2506 (3)	0.48894 (15)	0.0245 (4)	
H12	−0.003 (2)	0.321 (4)	0.538 (2)	0.041 (7)*	
C13	0.77879 (19)	0.4440 (3)	0.6316 (2)	0.0334 (5)	
H13A	0.781 (2)	0.381 (4)	0.570 (2)	0.040 (7)*	
H13B	0.787 (2)	0.361 (4)	0.686 (2)	0.036 (7)*	
H13C	0.836 (3)	0.547 (5)	0.633 (3)	0.068 (11)*	

### 1-Benzyl-4-(methylsulfanyl)-3a,7a-dihydro-1H-pyrazolo[3,4-d]pyrimidine Atomic displacement parameters (Å^2^)

**Table:**

	*U*^11^	*U*^22^	*U*^33^	*U*^12^	*U*^13^	*U*^23^
S1	0.0236 (3)	0.0230 (3)	0.0341 (3)	0.00000 (17)	0.0021 (2)	−0.00033 (17)
N1	0.0225 (8)	0.0211 (8)	0.0327 (9)	0.0011 (6)	0.0039 (7)	0.0009 (6)
N2	0.0259 (9)	0.0198 (8)	0.0295 (9)	−0.0009 (6)	0.0042 (7)	−0.0002 (6)
N3	0.0194 (8)	0.0252 (9)	0.0260 (8)	−0.0017 (6)	0.0021 (6)	−0.0013 (6)
N4	0.0242 (9)	0.0251 (9)	0.0293 (8)	0.0022 (7)	0.0024 (6)	−0.0034 (7)
C1	0.0211 (9)	0.0214 (9)	0.0178 (8)	−0.0022 (7)	0.0006 (6)	0.0010 (7)
C2	0.0281 (11)	0.0191 (10)	0.0387 (12)	0.0024 (8)	0.0039 (9)	−0.0011 (8)
C3	0.0214 (9)	0.0242 (10)	0.0194 (8)	−0.0034 (7)	0.0018 (7)	−0.0006 (7)
C4	0.0227 (10)	0.0242 (11)	0.0281 (10)	0.0038 (7)	0.0023 (8)	−0.0031 (7)
C5	0.0224 (10)	0.0197 (9)	0.0216 (9)	−0.0003 (7)	0.0012 (7)	−0.0016 (7)
C6	0.0201 (9)	0.0320 (11)	0.0246 (9)	−0.0067 (8)	0.0045 (8)	0.0002 (8)
C7	0.0161 (8)	0.0200 (8)	0.0255 (9)	−0.0029 (7)	0.0036 (7)	0.0010 (7)
C8	0.0190 (10)	0.0216 (9)	0.0294 (10)	0.0004 (7)	0.0047 (8)	0.0002 (7)
C9	0.0302 (11)	0.0250 (10)	0.0272 (10)	−0.0050 (8)	0.0076 (8)	−0.0037 (7)
C10	0.0265 (10)	0.0329 (11)	0.0273 (10)	−0.0099 (8)	−0.0036 (8)	0.0048 (8)
C11	0.0164 (9)	0.0299 (11)	0.0400 (11)	−0.0013 (8)	−0.0008 (8)	0.0082 (9)
C12	0.0187 (9)	0.0219 (9)	0.0337 (10)	−0.0012 (7)	0.0066 (7)	0.0002 (7)
C13	0.0256 (11)	0.0296 (12)	0.0441 (13)	−0.0020 (8)	−0.0003 (9)	0.0039 (9)

### 1-Benzyl-4-(methylsulfanyl)-3a,7a-dihydro-1H-pyrazolo[3,4-d]pyrimidine Geometric parameters (Å, º)

**Table:**

S1—C1	1.7400 (19)	C6—H6A	0.95 (3)
S1—C13	1.789 (2)	C6—H6B	0.93 (3)
N1—C1	1.337 (3)	C7—C12	1.390 (3)
N1—C2	1.358 (3)	C7—C8	1.394 (3)
N2—C2	1.313 (3)	C8—C9	1.388 (3)
N2—C3	1.350 (3)	C8—H8	0.91 (2)
N3—C3	1.346 (3)	C9—C10	1.388 (3)
N3—N4	1.370 (2)	C9—H9	0.90 (3)
N3—C6	1.466 (2)	C10—C11	1.385 (3)
N4—C4	1.320 (3)	C10—H10	0.83 (3)
C1—C5	1.396 (3)	C11—C12	1.392 (3)
C2—H2	0.97 (3)	C11—H11	0.86 (3)
C3—C5	1.406 (3)	C12—H12	0.97 (3)
C4—C5	1.426 (3)	C13—H13A	0.96 (3)
C4—H4	0.89 (2)	C13—H13B	0.95 (3)
C6—C7	1.510 (3)	C13—H13C	1.02 (4)
			
C1—S1—C13	101.59 (10)	C7—C6—H6B	111.9 (15)
C1—N1—C2	117.19 (17)	H6A—C6—H6B	106 (2)
C2—N2—C3	111.93 (17)	C12—C7—C8	119.25 (18)
C3—N3—N4	110.87 (16)	C12—C7—C6	120.62 (17)
C3—N3—C6	127.36 (18)	C8—C7—C6	120.13 (17)
N4—N3—C6	121.74 (16)	C9—C8—C7	120.25 (19)
C4—N4—N3	107.11 (16)	C9—C8—H8	119.2 (14)
N1—C1—C5	120.45 (17)	C7—C8—H8	120.5 (14)
N1—C1—S1	118.78 (15)	C10—C9—C8	120.03 (19)
C5—C1—S1	120.76 (15)	C10—C9—H9	121.0 (18)
N2—C2—N1	129.09 (19)	C8—C9—H9	119.0 (18)
N2—C2—H2	115.3 (18)	C11—C10—C9	120.2 (2)
N1—C2—H2	115.6 (18)	C11—C10—H10	123 (2)
N3—C3—N2	126.83 (18)	C9—C10—H10	117 (2)
N3—C3—C5	107.31 (18)	C10—C11—C12	119.65 (19)
N2—C3—C5	125.86 (18)	C10—C11—H11	119.8 (18)
N4—C4—C5	110.24 (18)	C12—C11—H11	120.5 (18)
N4—C4—H4	119.6 (15)	C7—C12—C11	120.61 (19)
C5—C4—H4	130.1 (15)	C7—C12—H12	121.6 (17)
C1—C5—C3	115.47 (18)	C11—C12—H12	117.8 (17)
C1—C5—C4	140.06 (19)	S1—C13—H13A	109.4 (17)
C3—C5—C4	104.47 (17)	S1—C13—H13B	107.5 (17)
N3—C6—C7	112.75 (15)	H13A—C13—H13B	113 (3)
N3—C6—H6A	105.8 (16)	S1—C13—H13C	106 (2)
C7—C6—H6A	112.1 (15)	H13A—C13—H13C	105 (3)
N3—C6—H6B	107.8 (15)	H13B—C13—H13C	115 (3)
			
C3—N3—N4—C4	−0.3 (2)	N3—C3—C5—C1	−179.69 (16)
C6—N3—N4—C4	−178.65 (17)	N2—C3—C5—C1	0.2 (3)
C2—N1—C1—C5	−0.5 (3)	N3—C3—C5—C4	−0.1 (2)
C2—N1—C1—S1	−179.60 (15)	N2—C3—C5—C4	179.84 (18)
C13—S1—C1—N1	−0.32 (18)	N4—C4—C5—C1	179.4 (2)
C13—S1—C1—C5	−179.38 (17)	N4—C4—C5—C3	−0.1 (2)
C3—N2—C2—N1	0.3 (3)	C3—N3—C6—C7	−99.9 (2)
C1—N1—C2—N2	0.2 (3)	N4—N3—C6—C7	78.2 (2)
N4—N3—C3—N2	−179.71 (18)	N3—C6—C7—C12	−108.9 (2)
C6—N3—C3—N2	−1.4 (3)	N3—C6—C7—C8	71.1 (2)
N4—N3—C3—C5	0.2 (2)	C12—C7—C8—C9	−0.2 (3)
C6—N3—C3—C5	178.49 (16)	C6—C7—C8—C9	179.77 (18)
C2—N2—C3—N3	179.36 (19)	C7—C8—C9—C10	0.1 (3)
C2—N2—C3—C5	−0.6 (3)	C8—C9—C10—C11	0.0 (3)
N3—N4—C4—C5	0.2 (2)	C9—C10—C11—C12	0.0 (3)
N1—C1—C5—C3	0.3 (3)	C8—C7—C12—C11	0.2 (3)
S1—C1—C5—C3	179.39 (13)	C6—C7—C12—C11	−179.80 (18)
N1—C1—C5—C4	−179.1 (2)	C10—C11—C12—C7	−0.1 (3)
S1—C1—C5—C4	0.0 (3)		

### 1-Benzyl-4-(methylsulfanyl)-3a,7a-dihydro-1H-pyrazolo[3,4-d]pyrimidine Hydrogen-bond geometry (Å, º)

*Cg*3 is the centroid of the C7–C12 phenyl ring.

**Table:**

*D*—H···*A*	*D*—H	H···*A*	*D*···*A*	*D*—H···*A*
C2—H2···S1^i^	0.97 (3)	2.81 (3)	3.781 (2)	174 (2)
C13—H13*C*···*Cg*3^ii^	1.02 (4)	2.49 (4)	3.455 (2)	157 (3)

Symmetry codes: (i) *x*, *y*−1, *z*; (ii) −*x*+1, −*y*+1, −*z*+1.
